# Consumption: How to Prevent It and How to Live with It

**Published:** 1892-06

**Authors:** 


Consumption: How to Prevent It and How to Live with It. Its Nature, its Causes, its Prevention, and the Mode of Life, Climate, Exercise, Food, and Clothing, necessary for its Cure. By N. S. Davis, Jr., A. M., M. D., Professor of the Principles and Practice of Medicine, Chicago Medical College; Physician to Mercy Hospital; Member of the American Medical Association, Illinois State Medical Society, Chicago Medical Society, Chicago Academy of Sciences, Illinois State Microscopical Society; Fellow of the American Academy of Medicine; author of a hand-book on Diseases of the Lungs, Heart, and Kidneys. Philadelphia and London: F. A. Davis, publisher. 1891.

The title of this book is somewhat striking, and we confess that we do not understand its full import. It would be a boon to humanity if it could be taught how to prevent the disease in question so that it might not slay its hundred thousand annually in the United States. If Dr. Davis will tell the profession something that it does not already understand about this baneful malady, he will confer a lasting favor upon the human race. We discover in this book the usual rules for hygienic management, and some careful and considerate remarks with reference to the influence of properly selected climate. We think the best part of the book is contained in Chapter III., where some concise and well-considered rules are given, looking to the prevention of the disease, assuming that it is an infectious malady. The author has told much in this chapter that will serve to limit its ravages, if the rules that he lays down are adhered to. We commend the book to all who would read an agreeably written little volume on a subject of great concern to humanity.

